# Optimizing Telehealth for Diabetes Management in the Deep South of the United States: Qualitative Study of Barriers and Facilitators on the Patient and Clinician Journey

**DOI:** 10.2196/43583

**Published:** 2024-01-01

**Authors:** Alessandra N Bazzano, Tejal Patel, Elizabeth Nauman, Dana Cernigliaro, Lizheng Shi

**Affiliations:** 1 Department of Social, Behavioral, and Population Sciences Tulane University School of Public Health and Tropical Medicine New Orleans, LA United States; 2 Louisiana Public Health Institute New Orleans, LA United States; 3 Public Health Innovation and Action New York, NY United States; 4 Department of Health Policy and Management Tulane University School of Public Health and Tropical Medicine New Orleans, LA United States

**Keywords:** telemedicine, diabetes mellitus, chronic disease, patient participation, delivery of health care, COVID-19, mobile phone

## Abstract

**Background:**

The Deep South of the United States, and Louisiana in particular, bears a greater burden of obesity, diabetes, and heart disease compared with other regions in the United States. Throughout the COVID-19 pandemic, there has been a substantial increase in telehealth visits for diabetes management to protect the safety of patients. Although there have been significant advancements in telehealth and chronic disease management, little is known about patient and provider perspectives on the challenges and benefits of telehealth visits among people living with diabetes and providers who care for patients with diabetes in Louisiana.

**Objective:**

This study aimed to explore barriers, facilitators, challenges, and benefits to telehealth for patients with diabetes and health care providers as they transitioned from in-person to remote care during the early COVID-19 pandemic to understand potential optimization.

**Methods:**

A total of 24 semistructured qualitative interviews were conducted with 18 patients living with diabetes and 6 clinicians who served patients with diabetes to explore their experiences and perceptions of telehealth services for diabetes care. Approximately half of the participants identified as Black or African American, half as White, and 75% as female. Interviews were recorded, transcribed, and coded by experienced qualitative researchers using inductive and deductive techniques. A narrative, descriptive approach to the patient and clinician journey framed the study, including the development of internal journey maps, and reflexive thematic analysis was applied to the transcripts, with special attention to barriers and facilitators.

**Results:**

In total, 5 themes illustrated barriers and facilitators for participants: convenience, safety, and comfort are the benefits of telehealth for patients and clinicians; yet telehealth and in-person visits are valued differently; the convenience of telehealth may have a downside; technology acts as a double-edged sword; and managing expectations and efficiency of the visit experience was an important factor. Individual experiences varied in relation to several factors, including comfort level and access to technology, health system protocols for providing telemedicine, and level of diabetes control among patients.

**Conclusions:**

Recommendations for optimization include providing support to help guide and inform patients about what to expect and how to prepare for telehealth visits as well as allowing clinicians to schedule telehealth and in-person visits during discrete blocks of time to improve efficiency. Further research should address how hybrid models of telehealth and in-person care may differentially impact health outcomes for patients with diabetes, particularly for people with multiple chronic conditions in settings where access to technology and connectivity is not optimal.

## Introduction

### Background

In the United States, telehealth services have significantly advanced during the last 20 years, including in the Deep South, where high rates of cardiometabolic disorders, such as obesity [1], diabetes [2], and heart disease [3], are prevalent. The growth of new technologies and the high rates of internet and electronic device use in the United States have enabled telehealth to flourish—between 2010 and 2017, the percentage of US hospitals using technology to connect with patients increased from 35% to 76% [4]. With the COVID-19 pandemic, the role of telehealth has become even more important, providing care to those who need it without in-person visits that could put patients at increased risk of exposure. During the initial peak of the COVID-19 pandemic, telehealth visits between 2019 and 2020 increased from <1% to as high as 80% in places with high COVID-19 prevalence [5]. Although the increase in telehealth visits was substantial, disparities among patient subgroups remained prevalent, with video telehealth rates lowest among populations without a high school diploma; among adults aged ≥65 years; and among Asian, Black or African American, and Latine individuals [5].

This significant increase and expansion of telehealth services has been especially important for patients with chronic diseases, such as diabetes, which requires consistent management by health care providers to prevent serious complications and mortality [6]. Diabetes affects an estimated 34.2 million people or 10.5% of the US population. For adults, there are significant disparities, with the highest prevalence of diabetes among minoritized populations, those without high school education, and populations that live below the federal poverty level [7]. Diabetes is one of the leading causes of death in Louisiana, with approximately 14.2% of the population being diagnosed with diabetes [7]. People living with diabetes, particularly those who also have other health conditions, are at an increased risk for serious complications due to COVID-19 and therefore may benefit from remote care [8]. Telehealth visits likely reduce the risk of exposure to COVID-19 infection, as patients do not have to interact with others in waiting rooms, examination rooms, and health care facilities, while also avoiding the potential risk of exposure during transportation.

The potential benefits of telehealth services can be seen most acutely in states that bear the greatest burden of COVID-19 prevalence. Early in the pandemic, Louisiana experienced the fastest growth rate of COVID-19 infections in the world, with 2 parishes comprising the New Orleans metro area exhibiting one of the highest per capita death rates among metropolitan cities in the United States [9]. There were significant racial disparities in death rates in Louisiana at a magnitude greater than other epicenters of the pandemic in the United States [10], particularly among the Black or African American population, where the rate of death due to COVID-19 was significantly higher than that of other races [10]. In March 2020, the Louisiana Department of Health directed health care providers to postpone care for 30 days and encouraged providers to use telehealth services. At the same time, the Louisiana Department of Health eased Medicaid billing restrictions on the provision of telehealth visits, expanding the reach for patients who need care [11,12]. This paved the way for the expansion of and increase in telehealth services in Louisiana.

With the significant increase and continuation of telehealth visits during the COVID-19 pandemic and evidence that telehealth programs can be an effective way of managing diabetes compared with face-to-face care [13], a better understanding of both patient and provider perspectives is required to identify opportunities to understand and improve the role of telehealth in patient care for this condition. A recent qualitative study of technology use for diabetes care assessed patient perspectives, indicating a good potential for benefit, with some hesitation on the part of patients about the use of technology [14]. With the rapid transition to telehealth early in the COVID-19 pandemic, remote services have been shown to be well received and result in positive encounters despite technical challenges [15,16]. From the health care provider’s perspective, the advantages of telehealth included increased usage of services [17], improved provider well-being [17,18], accommodation of patients who face challenges for in-person care [18,19], and fostering a sense of family-centered care [18]. The disadvantages from the clinician perspective include issues with technology and disparities in patient access to technology [17-20], less personal connection [19], inability to complete in-person diagnostic tests [20], reimbursement complications and out-of-state licensure restrictions [17], and a preference for in-person visits for patient populations that would benefit more from in-person care [17]. Studies exploring patient perspectives have reported that benefits, such as reduced travel time, shorter wait time, and cost savings [15,21], as well as convenience and safety [15,22], were seen as advantages, whereas internet issues, technical barriers [21,22], lack of connection with their provider, and unfamiliarity with the telehealth process [22] were seen as barriers. One study of patient perspectives on cardiology telehealth found that although both in-person and telehealth visits were viewed favorably, patient satisfaction was rated slightly higher for in-person visits. They found that the domain of clinical competence had the only lower mean score for telehealth [21]. A recent study highlighted racial disparities in telehealth use in the South with the transition to telehealth during the COVID-19 pandemic, finding an increase in the proportion of female and Black and Hispanic patients. However, discrepancies were observed in the likelihood of using an audio-video telehealth service, with older, Black, urban, and Medicaid or Medicare insurance carriers less likely to use audio-video telehealth services [23].

Although there have been significant advancements in telehealth and chronic disease management, they have not yet met their potential for improving and addressing the needs of all patients equally. The demographic context of patients with chronic diseases, such as diabetes, may be misaligned with access to effective telehealth modalities, such as video visits [23]; thus, to investigate broader issues around the value of telehealth, we applied qualitative methods to an exemplar population of people living with diabetes.

### Objectives

This study was nested within a larger natural experiment study [24-28] and comprised the qualitative component of a mixed method approach that aimed to explore barriers, facilitators, challenges, and benefits to telehealth for patients with diabetes and health care providers in Louisiana as they transitioned from in-person to telehealth care following the COVID-19 pandemic. Data from this study complement the larger study’s quantitative examination of facilitators and barriers to the uptake, adoption, and implementation of telehealth services among Medicare patients with diabetes from the perspectives of health systems, health care providers, and patients. The larger study provided quantitative comparison of diabetes control and continuity of care between patients with and without telehealth use during COVID-19, providing a mixed method approach alongside the qualitative data presented here [28]. This study also aimed to provide insights into the broader context of telehealth value for patients with chronic diseases in lower socioeconomic contexts.

## Methods

### Overview

This study is a part of the larger Louisiana Experiment Assessing Diabetes Outcomes study, which aimed to assess the reach, effectiveness, adoption, implementation, and maintenance of chronic care management services. The details of the larger study can be found elsewhere [28]. This qualitative study included individual interviews with 18 patients with diabetes and 6 clinicians who provided diabetes care, who participated in semistructured interviews to explore their experiences, including barriers and facilitators to engaging in telehealth services for diabetes care. To guide the preparation of this study, the Standards for Reporting Qualitative Research Reporting Guidelines [29] were used at each stage of the process.

### Sampling and Recruitment

Purposive sampling was used to identify participants who were either patients who received a telehealth visit for diabetes care after March 1, 2020, or health system staff who had conducted or coordinated such visits. Recruitment of patients occurred through patient portal messages and secure emails, with phone call follow-up, and participants were purposively selected to represent the broader population of patients with diabetes in Louisiana who depend on Medicare and a mix of insurance providers. Chain referral sampling was also used, and interviewees from the previous year of the study assisted in the identification of potential participants. In total, 60 patients were invited and 18 of them agreed to participate, and among 10 clinicians who were invited, 6 agreed to participate, fulfilling the expectation of a sample sufficient for qualitative research using the principles of code saturation in data analysis [30,31]. Health care provider participants were defined as clinicians and included primary care physicians, endocrinologists, and advanced practice nurses, allowing for a variety of practice experiences. Clinicians were invited via email or phone to participate in interviews and given information about the study. The study steering committee, including patient partners and researchers, reviewed and facilitated participant recruitment strategies.

### Data Collection Methods

Interview guides were used to support the semistructured interviews and were developed in partnership with study steering committee members. To strengthen the validity, the guide was pretested with patient partners who had diabetes and experienced telehealth services during the pandemic. Interviews were conducted and recorded on receiving participant consent through videoconferencing for participants who stated that the technology was available to them and via telephone for those who preferred not to use videoconferencing. No patients had to travel for the interview, and all phone or videoconference interviews were conducted in private locations. Patient and provider participants were interviewed between October 2020 and April 2021 by researchers with advanced degrees in public health and experience in qualitative research. To acknowledge their contributions, participants received gift cards. A study description explaining the purpose of the research, goals for the interview, and participants’ rights was provided before the interviews, at which only the researchers and participants were present, and informed consent was obtained.

### Data Analysis

All data were securely stored on encrypted computers and accessed only by the researchers who completed the analysis. A narrative descriptive approach provided a guiding orientation to the analysis, with special attention paid to the participant journey and attendant barriers and facilitators. The study researchers have used reflexive thematic analyses [32]. A total of 2 experienced qualitative researchers collaborated in coding, and through inductive and deductive strategies, they proceeded to creation of categories and candidate themes from the interview transcripts. Open coding was used to describe candidate themes, whereas deductive coding was used to check themes against the data. Journey maps were also developed with human-centered design tools applied to interview data to explore barriers and facilitators, paint points, and high points on participants’ journeys with telehealth. During the analysis process, peer debriefing and reflexive journaling through memos were conducted. NVivo qualitative analysis software (QSR International) was used to manage data. Preliminary and final themes and analysis progress were shared with the study leaders and researchers, partner organizations, and patient stakeholders to receive feedback on the findings. The researchers discussed preliminary data during the review process and consulted with patient partners for validity.

### Ethics Approval

Institutional review board approval was provided for this research by Tulane University under reference number 906810.

## Results

### Participant Characteristics

A total of 18 patients with diabetes and 6 health care providers were interviewed for this study. Patient participants were predominantly female (14/18, 78%) and married (8/18, 44%). The average age of participants was 60 (SE 3.0; range: 35-78) years, and, on average, patients had been living with diabetes for 13 to 14 (SE 13.5; range 1-48) years at the time of study. Half of the patient participants described themselves as Black or African American (9/18, 50%) and half described themselves as White (9/18, 50%). Table 1 presents an overview of the participant demographic data. For participating health care providers, the average age was 50 (SE 5.1; range 38-75) years, and they had been practicing in their profession for an average of 20 (SE 5.8; range 7-46) years. The participants included primary care physicians, endocrinologists, and advanced practice nurses, allowing for a variety of practice experiences. A total of 4 provider participants identified themselves as female and 2 identified themselves as male. In addition, 2 providers identified as Black or African American, 1 identified as South Asian, 2 identified as White, whereas 1 provider participant did not disclose this information.

Most patient participants had Medicaid or Medicare health insurance coverage. Almost all participants lived within a 45-minute drive of their health care provider or hospital, except 1 rural residing participant who lived 4 hours away from their provider. The participants described managing several other health conditions in addition to diabetes, including asthma, overweight or obesity, heart conditions, thyroid disease, breast cancer, vision issues, sleep apnea, kidney disease, genetic disorders, and most commonly high blood pressure. Most participants identified as independent and self-sufficient, although many had friends and family members who checked in with them and ensured that they were managing their health and helped with transportation, technology, or advice on diet and lifestyle.

In terms of previous experience with telehealth, patient participants had an average of 2 to 3 previous telehealth visits (range 1-5 visits). Some participants reported using the phone for telephonic visits (8/18, 44%), whereas others reported using the telephone and video (10/18, 56%) for telehealth visits. Of 18 patients, 14 (78%) had returned to mainly in-person care at the time of the study while continuing to have some telehealth visits. Table 2 illustrates the participants’ telehealth usage patterns.

**Table 1:** Participant characteristics.

Characteristics^a^		Patient participants (n=18)	Provider participants (n=6)
Age (y), mean (SE; range)		60.39 (3.03; 35-78)	50.16 (5.08; 38-72)
Years living with diabetes (patients only), mean (SE; range)		13.53 (3.56; 1-48)	N/A^b^
Years in clinical practice (providers only), mean (SE; range)		N/A	20.08 (5.84; 7-46)
**Sex, n (%)**			
	Female	14 (78)	4 (67)
	Male	4 (22)	2 (33)
**Race, n (%)**			
	Black or African American	9 (50)	2 (33)
	South Asian	0 (0)	1 (17)
	White	9 (50)	2 (33)
**Marital status, n (%)**			
	Married	8 (44)	—^c^
	Divorced	4 (22)	—
	Single	4 (22)	—
	Widowed	2 (11)	—

^a^Characteristics, including race, refer to categorizations used by the United States Census Bureau and other entities; these are not an indicator of biological difference but are presented to provide context about socially constructed experiences.

^b^N/A: not applicable.

^c^Not available.

**Table 2:** Patient telehealth use characteristics (n=18).

Visit details and characteristics			Values
Telehealth visits, mean (range)			2.35 (1-5)
**Mode of visit, n (%)**			
	Telephone only	8 (44)	
	Telephone and video	10 (56)	
**Returned to in-patient care, n (%)**			
	No	4 (22)	
	Yes, with some telehealth	14 (78)	

### Overview of the Telehealth Journey for Patients and Clinicians

Both the patient and provider participants were asked to describe their user journey during the telehealth process. For patients, the activities for scheduling and accessing telehealth visits were generally similar across the different health systems they used. Participants typically described receiving a call or notification at least 1 day before their scheduled visit and receipt of a link to log into the visit. If the patient had trouble navigating the log-in process, some participants described that a member of the health care staff would reach out to help guide them. On average, telehealth visits lasted between 10 and 20 minutes. However, the reported duration of the full range of visits was between 1 minute for a patient who did not have results to discuss to a full hour for a participant who had a more complex health issue to discuss. Patients used phones and laptops to connect to visits, and video or phone visits were often conducted at home (locations within the home included kitchens, offices, living rooms, bedrooms, and bathrooms), but some took place in a car or outside the home. Where patients took telehealth visits at home, they also reported that family members might be at home or in the same room as the patient during the telehealth visit.

From the health care provider perspective, the telehealth process varied based on the standard operating procedures of the individual health system. Health systems had different scheduling approaches for telehealth; some allocated full days or half days for scheduling strictly telehealth visits, with in-person appointments scheduled during a different block of time; others had both in-person and telehealth visits interspersed throughout the day on their schedule. The latter format was considered the least efficient by clinicians due to longer wait times when switching between in-person and telehealth visits, as well as difficulties making connections with patients. Health care providers reported variable visit durations for telehealth visits between 10 and 20 minutes on average, and these were taken from different locations. Certain facilities had physician workrooms, conference rooms, or offices that the provider could use. A couple of providers reported taking calls from home; however, challenges with this approach were mentioned, including distractions. Clinicians described using smartphones, laptops, and tablets for telehealth and also mentioned apps, such as Doximity, which conceals the phone number of the caller, as being used in telehealth care provision [27].

### Facilitators and Barriers to Telehealth Care for Diabetes

#### Overview

The 5 themes drawn from the qualitative analysis are presented in subsequent sections. Overall, participants described positive experiences with telehealth during the pandemic, although perspectives ranged from a strong preference for telehealth to a strong preference for in-person care. The participants described valuing the convenience, safety, and comfort of telehealth visits. However, patients seemed to value telehealth visits differently than in-person visits, indicating the importance of in-person interactions with their provider and physical check-ups. Furthermore, health care providers stated that patients had different expectations for a telehealth visit and that sometimes the flexibility and convenience of the telehealth visit translated to patients not being prepared for visits, having unrealistic expectations about wait times in telehealth, and taking calls from locations that were inappropriate for health care (eg, while walking through a retail store).

The role of technology as both a barrier and a facilitator was a key theme for both patients and clinicians. Clinicians who experienced it as most beneficial were typically more comfortable navigating technology, had more experience with telehealth, or were part of a health system that provided support around technology. Patients with diabetes who were more comfortable with technology used with telehealth (eg, those who could easily navigate devices, log into portals for video telehealth visits, and receive visit summaries from the patient portal) described the most positive experience with telehealth visits.

Clinicians described valuing the convenience and safety of telehealth for patients, particularly the ability of patients to check in with them without traveling to a health care facility, the efficiency of the system for those who were tech savvy, and improved safety for patients with diabetes who were at high risk for severe COVID-19. Similar to patient participants, clinicians described challenges and delays in care because of issues with technology. For providers, telehealth was perceived to be the best option for patients with controlled diabetes. Both clinicians and patients endorsed a preference for a hybrid model consisting of both in-person and telehealth care.

#### Convenience, Safety, and Comfort of Telehealth for Patients and Providers

Patients described many facilitators for using telehealth, with the most common being convenience, particularly for those with mobility issues, those with busy schedules, or those who lived at greater geographical distances from their provider. Patients described valuing the convenience and comfort of not having to drive, park, check in, and wait for their appointment as well as the ease of talking to their provider over the phone:

Yes, I love [telehealth visits] I don’t have to get dressed, I don’t have to drive, I don’t have to do nothing, but answer my phone...That’s a big help, you don’t have to get dressed to go out and you don’t have to spend no money on no gas. It’s just worth [it] all around. You can definitely be safe because you are not going into no crowd.

Black or African American female patient aged 77 years, living with diabetes for 11 years

If I’m too busy, I'd rather do telehealth than trying to finagle my schedule. “Oh, I got to go to the doctor. I got to do this. I got to do that.” Telehealth would be much easier. I probably could sit in the car, going wherever I'm going, and do my telehealth visit and still be at the place wherever I need to be.

Black or African American male patient aged 46 years, living with diabetes for 2 years

I feel like you still get the same healthcare as if you were standing there in front your doctor, unless they have to look at something, like your wound or something ...i think it is very accessible and very easy to use.

White female patient aged 43 years, living with diabetes for 8 years

One participant’s view was that she felt more honest and open sitting at home, speaking with her provider during a telehealth visit:

I think I'm more honest when I'm sitting in my chair at home. It's harder for them to judge me virtually, in my mind, at least. I think I'm just so much more comfortable here, that I'm more open.

White female patient aged 60 years, living with diabetes for 20 years

Participants also discussed valuing their safety. Patients who did not have a strong preference for in-person or telehealth visits mentioned that they would do whatever was the safest and recommended during the pandemic. Although an in-person visit was discussed as important if they had a physical issue they felt their physician should assess, patients acknowledged that traveling and being in a hospital could put them more at risk for COVID-19 infection:

Either way it's fine with me, but for me, the virtual is the best for everybody's sake. I believe that's the best way to do it. I’m fine with it. A lot of other people are not.

Black or African American male patient aged 69 years, living with diabetes for 18 years

No. I think [I don’t have a preference for] either one, I mean, I like the convenience of not having to go into the office, especially right now having COVID and then hearing that I could get it again, the safety part of it.

White female patient aged 43 years, living with diabetes for 8 years

One thing I did like, I was at my house. I didn’t have to go through all the shenanigans at the hospital with checking in and all this kind of stuff and social distancing stuff, worrying about blood pressure. Some people social distance, some people don’t. I didn’t have to be bothered with that.

Black or African American male patient aged 46 years, living with diabetes for 2 years

I liked it because my doctors know I’m taking my medicine and I like him because he has a pleasant spirit. I don’t like driving there and finding parking. I didn’t feel rushed. I would do a virtual visit, I’m nervous of the virus so I don’t want to go in the office.

Black or African American female patient aged 66 years, living with diabetes for 18 years

Health care providers also mentioned safety from COVID-19 as a factor in telehealth visits. This was especially important for providers who worked in locations where it was difficult to physically maintain the distance between patients or in crowded waiting rooms. In such cases, the risk was perceived as especially high, particularly for patients with multiple conditions or those who were not vaccinated:

I don’t think it's really fair or safe, [for] the patients who aren't vaccinated to come in and sit in a small lobby early with patients who have diabetes and the like. I just don’t think it's good public health. You choose not to be vaccinated then you’re probably going to get a telehealth or televideo call...I'm excited about that.

Health care provider aged 75 years, practicing for 46 years

Other facilitators for telehealth included patients feeling that their telehealth visits provided sufficient time with their provider and that their provider was attentive and took an appropriate amount of time with the telehealth visits:

I liked that I didn’t have to go out there, and still I felt like I was right there talking to him. He is tall and big and younger than me and just like a sweet teddy bear not really overweight just like a teddy bear. He was a doctor that I could sit and talk to him and he would listen. It took me years to find him.

White female patient aged 75 years, living with diabetes for about 10-15 years

It works fine for me. I feel like my doctor is very attentive and I can ask any questions, so it works fine either way for me.

Black or African American female patient aged 51 years, living with diabetes for 7 years

I didn’t feel [rushed]. Everything was answered. Everything was fine as if I was in the office because I got my instructions on what I needed to do with my blood work. It was just fine.

Black or African American female patient aged 48 years, living with diabetes for 2 years

Participants also mentioned the comfort and ease of telehealth visits. One participant mentioned that given the ease of a telehealth visit, she was more inclined to call the physician, knowing she would not have the hassle of driving in:

Because it’s so much easier for me to be able to do one of these visits just over my phone from home, you know that sometimes, there’s something that goes wrong, you think, “Oh, I should probably go to the doctor, but I don’t want to mess with it”? I think I would be more inclined to go ahead and contact the doctor knowing that I wouldn’t have to go in.

White female patient aged 60 years, living with diabetes for 20 years

A patient reported that their telehealth visit allowed them to be prepared and enabled a thorough assessment, especially as it was the first visit:

It allows me to go ahead and have my questions prepared to raise the questions that I need to raise. They go through reports...with the first virtual visit that I had [with my doctor], I almost died. It was 45 minutes long. [That’s] the thing my son said was good about it is that he was here, so he heard everything.

White female aged 78 years, living with diabetes for about 45 years

#### Valuing Telehealth Differently From In-Person Visits

Although the patient participants described appreciating the convenience, safety, and comfort of telehealth visits, a model of care that included both in-patient and telehealth visits was considered optimal. One caveat is the need for laboratory tests, such as hemoglobin A_1c_ (HbA_1c_), for which many patients underwent laboratory work at outside facilities before their telehealth visits. Patients who had difficulty uploading previously obtained laboratory results and navigating technology preferred in-person care:

[The telehealth visit] was good, but I told him I couldn’t wait to see him. I would rather go into the office. It was alright, but I like when I go to see him. He’s a very patient doctor the [telehealth] conversation didn’t feel rushed, I just think in person was better.

Black or African American female patient aged 67 years, living with diabetes for 7 years

I would use [telehealth] in the future unless I was having problems, like if I had other issues like high blood pressure or something like that. I wouldn’t use it all the time, but to be convenient like, if I just needed a prescription refill, the telehealth visit would be fine for me.

Black or African American female patient aged 48 years, living with diabetes for 2 years

In addition to endorsing the importance of a physical check, participants wondered about the billing associated with telehealth visits. One patient described this as follows:

Well, virtual—I didn’t get any information, no blood tests and there's no A1C. It was a pointless endeavor. It was just [hospital] saying, “We can’t figure this out. We're just going to do a pretend appointment so we can bill the insurance company.” It was a phone call, so I had the phone in my hand, I’m in my room by myself. They asked me how I'm doing, I said fine and that was the end of it. Probably less than a minute.

White male aged 70 years, living with diabetes for 3 years

For clinicians, telehealth visits were generally described as a good asset for diabetes patient care. Provider sentiment was that telehealth worked best as an additional element for patients but could never fully replace in-person care. Similar to patients, clinicians discussed the convenience of telehealth visits, especially for patients who lived in rural areas, had issues with transportation, or were older adult patients who have mobility challenges:

I think probably 80% of patients are fine. We could do this over the video or no video or phone, not in person, let's say. Unless you have open wounds or unless there's changes if we had seen you before, and you’re saying, “Well, Doc, everything's similar.”...I think probably majority is fine. Not to say we should convert all of their visits to tele-visits, but possibly two out of three, or every other, or some, whatever is convenient.

Female health provider aged 38 years, practicing for 7 years

In certain circumstances, providers could not substitute a telehealth visit for an in-person visit, such as with a new patient, patients with multiple chronic conditions, where a patient had major changes in their health status, or where a patient had symptoms of uncontrolled diabetes. One provider noted difficulty in seeing patients who were not English speaking for telehealth visits, as it was challenging to get an interpreter for a telehealth call. A physician described the need to see new patients in person as follows:

I'm personally seeing all my new people in person. If they were seen by another endocrine provider, or if they were a new referral, I see them the first time in person. I just feel more comfortable that way, knowing that I've examined them thoroughly. We have their vitals, we've gone through the meds. After that, I usually do follow up tele-visits.

Female health provider aged 40 years, practicing for about 5 years

Clinicians discussed telehealth as generally best for patients whose diabetes was well controlled as a check-in to ensure that patients were continuing to do well. Overall, however, providers would prefer to see patients in person if there were any issues or complications:

For the patients who are doing very well, who are largely on auto pilot, for whom there’s not very much to discuss...It's usually just, “Oh the labs look very good, just keep doing what you’re doing.” It works well, it works quite well, but for the patients who have more complexity, more comorbidities for some, there's a fair amount that needs to be done. You end the meeting feeling that there's still more that needs to be done. In some cases, you actually have to tell the patient that they do need to set up an appointment to be seen in person. It all depends on how much current comorbidity and how well controlled the patient is.

Male health care provider aged 53 years, practicing for 28 years

Given the benefits and minor drawbacks of telehealth visits for diabetes care, provider participants described telehealth as being a permanent part of patient care. One physician noted:

I really do think there’s definitely place for telemedicine for these patients even past pandemic. Maybe they come into the clinic once in six months or even once a year depending on how well their control is for diabetes. Of course, if they truly need to be seen, they need to be seen.

Female health provider aged 38 years, practicing for 7 years

#### Convenience of Telehealth May Have a Downside

A downside to the flexibility and convenience of telehealth visits may be that it also affects how well prepared patients are for a visit and lessens focus and attention on the visit content itself. As telehealth visits can be taken from a wide variety of locations, patients sometimes reported having their visits in places that were not private or where significant distractions prevented them from focusing on the visit. This was especially noted during telephonic visits.

One participant described taking a call from the car from the side of the road, whereas another reported taking a call while out shopping. Patients who were multitasking and engaging in driving or shopping could be less likely to have laboratory or blood glucose results available for the visit:

With me, it was fine the first time because after we talked on text, I didn’t have to leave work and go over there...I pulled over on the side of the road and we talked and after...I went on to my destination.

Black or African American male patient aged 69 years, living with diabetes for 18 years

I like the fact that even if I am at work or home, or even in the car, it’s just the flexibility, like, “Hey, if I can’t make it to the—If I couldn’t get to the doctor’s office, I could do it from wherever.”

Black or African American male patient aged 47 years, living with diabetes for 1 year

Although participants valued the comfort and convenience of telehealth visits, health care providers noted that they may not be as well prepared for such visits. One provider mentioned that some patients do not see the telehealth visit as a “real” physician’s visit, so they can be less prepared for the visit or in a location that is distracting:

I find patients are going about their day and then realizing their appointment is live and [saying] “I don’t have my data because I’m at Target.” They didn’t really think of it as a doctor’s visit and so they didn’t prepare as well as they normally would...so it’s a little bit distracting. They don’t have their pills with them or they are not at home to say, “Oh, I’m taking this much of that or this medicine or that medicine.”

Female health provider aged 38 years, practicing for 7 years

A clinic coordinator also noted that patients sometimes did not follow through laboratory tests in the context of telehealth visits, impacting their ability to follow-up:

The doctor does the telehealth visit, he put in his plan, he wants the patient to follow up in 3 months with labs. Then I mail the patient a reminder appointment, advise them, and get the labs done before their appointment. Once a patient clicks on for the visit, if they don’t do the lab—usually I prep the schedule before an appointment and be like “You didn’t do your lab?” That kind of thing. The patient being compliant is a big factor with a telehealth visit.

Male health provider aged 53 years, practicing for 23 years

When patients are on the go, the technology can also be less reliable, as noted by another clinician:

The negative side of [telehealth] is the internet connection...If the patient is not at home or a home setting, like if they are in a car, I find those patients won’t be able to get good reception.

Female health provider aged 38 years, practicing for 7 years

Clinicians also noted often having to wait on the line for patients to locate blood glucose logs or medicines. This situation was contrasted with in-person visits, for which patients know they are expected to arrive with their information. Where patients needed to find laboratory results or other information during a visit or call, it could significantly delay visit length having a knock-on effect for subsequent telehealth patients:

With telehealth, usually when they come into the clinic, their vitals are done and the nurse reconciles their meds for them...Most people do have the equipment at home to check. It’s not really been an issue, but sometimes you have to wait for them to do it while you’re on the phone with them just to make sure that their blood pressure is okay. The same thing with medication, sometimes they don’t remember or they have to go check. You’re waiting a little bit for that to happen because you don’t have support of staff, and you’re doing it yourself, those things take longer sometimes.

Female health provider aged 40 years, practicing for about 5 years

#### Technology as a Double-Edged Sword

Comfort level and proficiency with technology were variable among both patients and health care providers, as were the different modes and platforms used by health systems to provide telehealth visits.

Some patients were only comfortable using their phones for a visit, whereas others had no trouble navigating video telehealth visits accessed through a patient portal. Those who felt most comfortable with technology were more amenable to telehealth as part of their care and seemed to have a better and more efficient experience. One patient described this as follows:

No, [no difficulties with the technology]. I was fortunate enough, we had been using that type of communication at work, so I was a little familiar with the process, how it works. No, I didn’t have any issues with it at all.

White male patient aged 57 years, living with diabetes for 1 year

However, navigating technology for many patient participants was an issue. Depending on their technological capabilities and schedules, patients preferred either phone or video visits. Patients who were less technologically savvy described frustrations navigating the system as well as getting used to the telehealth visit experience and were more likely to value in-person care. Some participants mentioned having help from family members or having someone from the hospital call before their first visit to ensure that they could log on; however, others did not have support with the technology. One participant mentioned the following:

I just think that when it’s your first time on there, if they actually had help for someone, an elderly person to do telehealth. For the first time, I think somebody might need to coach that person or assist that person logging on. Just make it friendlier for the elderly or the up in age people.

Black or African American female patient aged 46 years, living with diabetes for 2 years

I was a little concerned for the very first time, I was like, “Oh, I hope I don’t have a problem with the phone, what if my video doesn’t work?” I had no problems from day one, everything worked out perfectly.

Black or African American female patient aged 51 years, living with diabetes for 7 years

The first time it was aggravating because I couldn’t make the [technology] connection, so they just got me later. Generally speaking, I prefer to go in person, but depending on which doctor and what reason [for it], telehealth is just fine.

White female patient aged 59 years, living with diabetes for about 15 years

From the clinician perspective, the specific technology, for example, the internet connection and how comfortable the patient was in navigating the connection, was mentioned as being very challenging. This was likely to be more of an issue for health systems primarily serving low-income patients. A clinician within a federally qualified health center described it as follows:

It was challenging, even to get them registered between the back and forth, we would have my medical assistant, myself and an IT person and the nurse, so you would have 4 people trying to launch one visit. It would take 45 min to do a back and forth...just trying to troubleshoot and talk a patient through on a landline, to help launch something on an iPad.

Female health provider aged 51 years, practicing for 22 years

One provider described how some lower-income patients had “Katrina phones,” which were provided just after Hurricane Katrina in 2005 and do not have video, voicemail, or text options, making it difficult to call back or get in touch with the patients. Other issues described were patients not answering a call from a phone number that they did not recognize or not being savvy with text or voicemail, which hampered scheduling or contacting patients:

Some patients have phones that they call Katrina phones. It was government- sponsored phones that don’t have voicemail, that have very few minutes and so…they either pick up or they don’t pick up. The other issues is when we call from the hospital, the phone [number] may be private...most of our patients…don’t answer any of those...Then some patients have a fancy phone…and they don’t really know how to use it...I think, definitely, a good portion of my population is not really tech-savvy. Again, I think training them to use this more and more it will work, it’s fine. It’s just we couldn’t make it all happen in a month.

Provider 2

There were technical issues going on that I think made [the telehealth process], particularly at the beginning, it wasn’t always very smooth and patients weren’t looking at times and everything. The elderly were having difficulty linking in.

Male health provider aged 75 years, practicing for 46 years

Telehealth visits were most beneficial and efficient when patients could navigate the system and upload their health information, such as blood pressure, glucose levels, and weight. When patients did not have this information available, telehealth visits were not as comprehensive:

Some patients don’t have access to take their own vital signs, like their blood pressure, pulse, they don’t [have] a pressure kit. When they log into the app, the link is going to ask them their weight, blood pressure, and if they have that available, they’re supposed to input it...some patients input their vitals some don’t.

Female health provider aged 44 years, practicing for 12 years

However, it was noted that as patients had more telehealth visits, the process improved. One clinician stated as follows:

We still have occasional glitches where you have a patient who is scheduled and signed up for a telehealth visit, and there’s trouble with them logging in. It’s a lot less now because people are becoming more accustomed. If they’ve done it once, they’ve done it twice, now it’s a matter of habit. They’re familiar with it now.

Female health provider aged 51 years, practicing for 22 years

#### Managing Expectations and Efficiency of the Visit Experience

Expectations about how efficient telehealth visits should be, including scheduling, logging in, and undertaking a visit, were frequently noted. On the one hand, patient expectations for timely and efficient care were not met, whereas on the other hand, care providers were limited by their ability to meet expectations based on how their schedules were set up and how their health system operated telehealth visits.

Patients sometimes described long wait times for health care providers to log into their telehealth visits when they had an appointment scheduled for a specific time. As telehealth calls can be taken from anywhere and had less of a wait time to schedule, there was a perception that there would not be a wait time for a visit. The participants were frustrated when these expectations were not met:

I did have a problem when it was time for my appointment because on the app, you have like a grey screen, and it stays like that for a while because you’re waiting on the doctor to come in, so I called him twice because the appointment was going over time. My appointment was for 10:00. It was 10:30 and nobody had came on the line. I thought I was doing something wrong or I thought something had happened or I missed the appointment.

Black or African American male patient aged 46 years, living with diabetes for 2 years

Providers noted that they could not meet patients’ expectations of efficiency because of scheduling and other issues that were outside their control. This contrasted with the expectation that patients had with in-person visits. One clinician described this as follows:

I think when a patient is in your office and they’re sitting for 20 minutes, it's expected my doctor is running late...Unfortunately, that's the reality. It shouldn't be that way, but that's how it is. On the phone, when you say I'm going to call around 11, they really think it's going to be exactly 11. You may be 11:30 just like you would be in clinic or even harder because you can’t get in touch with them themselves let alone the rest of your clinic flow.

Female health provider aged 38 years, practicing for 7 years

One patient participant described being in his car, not expecting to have to wait for the provider to log on. Participants mentioned that once they had 1 or 2 visits, the process became easier, and they knew what to expect:

I just assumed that I could [log in to telehealth] if I was out. I assumed that I can still have a telehealth visit where I was at, but I remember saying I can go sit in my car. I think it was going to be too long to sit in the car. It had to be rescheduled.

Black or African American female patient aged 48 years, living with diabetes for 2 years

Another participant waited 15 minutes to reschedule because of technical issues from the provider. They stated the following:

For me, it wasn’t hard, but I know on the other end, I don’t know if it’s because just they were just starting using it because there were a couple of times where I’ve called...I logged in for the telehealth visit and nobody was there and I waited 15 minutes and I said, “Either they are running over or they forgot, or something happened.” I log out, then I get a message saying, “Hey sorry, we were having technical difficulties. Can we reschedule for such and such a time?”

Black or African American male patient aged 47 years, living with diabetes for 1 year

Clinicians lamented the challenges and need to temper patient expectations of timeliness in telehealth scheduling:

If you had patients scheduled on half an hour visit, but it took you 45 minutes just to launch it, and then another 15 to 20 minutes on the visit, we would be behind. We’re trying to call the patient saying, “We’re sorry we’re late. Just give us a minute.” One patient could take upwards of of an our to do a 15 minute visit or a 20 minute visit.

Female health provider aged 44 years, practicing for 12 years

Yes, definitely, patients have expressed their frustration with, “Well, I thought they were going to call at 11 and it’s 11:30, or 40, or 20, whatever it may be.” I think they expect a little more easier contact just because it’s by the phone, and to us, it’s harder, I think.

Female health provider aged 38 years, practicing for 7 years

Scheduling issues and wait times were commonly discussed by health care providers as some of the biggest barriers to efficient care:

There are patients who they may get in 30 minutes before the appointment and then leave out 10 minutes before...and then we have to track them down and say, “You didn’t wait long enough...your appointment was at 2:00, you went in at 1:30 and left at 1:45.” Then they've lost the link...particularly at the beginning it wasn't always very smooth and patients weren't looking at times and everything.

Male health provider aged 75 years, practicing for 46 years

Clinician participants described challenges with scheduling in-person versus telehealth visits, which affected patient wait times and depended on how their health system organized the clinician workflow:

When we initially started, we had them interjected into the day and that’s when we realized the inefficiencies with getting the system to launch. We quickly transitioned to one or two, and each provider had their preference. I had a telehealth day. I had one day that was designated for telehealth and that's it. Then some providers they had a certain time frame designated for telehealth with maybe their mornings, they saw patients in the office, and then in the afternoon they would have a few telehealth scheduled.

Female health provider aged 51 years, practicing for 22 years

I try to group them all together. We’re going to do virtual in the morning and in-person evening. I don’t like to have a virtual and then in-person, because...you’re going to run late behind if you have an in-person patient and then you got a televisit. Because somewhere you might have went over with the in-person visit, and the patient is on to the televisit. Those kinds of situations make a doctor run late too.

Female health provider aged 44 years, practicing for 12 years

## Discussion

### Principal Findings

The study found that both patients and providers appreciated the flexibility and convenience of telehealth visits. However, telehealth visits were considered by clinicians to be best for those whose diabetes is well controlled or less complicated for patients who were more tech savvy and have internet connectivity and for those with transportation challenges. A caveat of telehealth visits was that patients seemed not to approach the visit with the same focus and preparation as an in-person visit—patients could be distracted, lack important information on their condition, or hold different expectations about the visit, such as short wait times. In addition, some patients felt that telehealth visits were not as comprehensive. A mixed model of both in-person and telehealth visits was considered optimal by most patients and providers of diabetes care.

A recent study in Missouri found that women, older patients, and those with Medicare, Medicaid, and self-pay statuses used telehealth more during the beginning of the COVID-19 pandemic, although older patients; Black patients; urban patients; and those with Medicare, Medicaid, and self-pay status were more likely to use phone only compared with audio-video telehealth services [23]. Our study sheds light on some of the nuances of the experiences of a similar population in the South, as our patient population identified as predominantly female, older, and using Medicaid or Medicare and half identified as Black.

Our results showed that overall, for both patients and health care providers, telehealth was viewed as an important part of diabetes management and care; however, a hybrid model incorporating both in-person and telehealth visits was considered optimal, as has been noted in other studies [15]. Despite issues with connectivity and scheduling complications, both patients and providers appreciated the flexibility and convenience of telehealth visits, as has been described in other studies [19,21,22,33]. However, from both client and clinician perspectives, telehealth visits were considered best for those whose diabetes was well controlled, for patients without complications, and for those who were tech savvy or have good internet or cellular connectivity, which echoes the findings from prior studies on telehealth [5,17,19]. The benefits of telehealth appear to be especially important for patients with diabetes who have difficulty traveling to see their physician, including older adult patients, with the caveat that they must also be comfortable navigating technology [33,34], and in particular, telehealth has been shown to be beneficial for patients in rural communities [35].

For patients with chronic diseases, such as diabetes, health management through telemedicine can be crucial for continuity of care and avoiding exposure to COVID-19 [6,36]. However, the convenience and flexibility of telehealth visits must be weighed against the perception that virtual care may be less comprehensive or effective. Health care providers in the study discussed how patients sometimes would not approach a telehealth visit with the same focus and preparation as an in-person visit. Motivation to continue engaging in diabetes care is another important consideration, as one study noted the importance of combining “eHealth” with regular face-to-face consultations to avoid reducing patient motivation for engagement [37]. There may be downsides to the flexibility and convenience of telehealth, as patients and providers described visits taking place in distracting environments, difficulty in managing expectations regarding wait times for visits, or lack of diagnostic information, such as available blood glucose results.

### Comparison With Prior Studies

Studies have indicated that most patients feel that telehealth visits can be as effective as in-person visits [16,38]. However, one study identified that patient respondents with higher HbA_1c_ levels who completed a telehealth visit were more likely to report that video care did not save them time, money, or stress, although a majority of those respondents still felt that video care was effective or beneficial in some way. In the same study, respondents with higher HbA_1c_ levels who had *not yet had* a video appointment were also more likely to report difficulty connecting remotely and were more concerned about the quality of care using video [15].

Technology was discussed by participants in this study as both a barrier and a facilitator for telehealth. Both patients and providers described a learning curve for using telehealth services. Older adults who were not as comfortable with technology seemed to prefer telephonic visits compared with video visits because of the ease of cell phone use, which has been noted in other studies [17,23,39,40]. Our study highlights some barriers to audio-video telehealth visits for this population, such as outdated technology (Katrina phones), inconsistent internet service, and lack of comfort with technology. Furthermore, age, sex, median household income, insurance status, and marital status have been found to be associated with patient participation in telehealth [41], providing evidence of structural inequality affecting patient comfort and the ability to engage in and benefit from telehealth. One study found that communities at greater risk of needing support after a disaster were significantly more likely to experience more barriers to telehealth, including access to reliable internet, low uptake of use of web-based medical portals, not feeling comfortable with technology, and more likely to report language barriers as a concern [42]. The Gulf South and Louisiana in particular are at high risk for similar events.

For patients, having the support to navigate early visits appeared to be important for improving the experience, and with more exposure to telehealth, the process became easier. Patients in this study had a broad range of experiences with telehealth (from 1 to 5 visits), which has been noted to impact perceptions of telehealth, particularly related to comfort with technology [35]. Issues and challenges with technology and computer literacy have been found to be significant barriers to the adoption of telehealth [43]. Similar hindrances have also been noted with other forms of non–face-to-face care in this setting [24,26], indicating that in all such remote clinical encounters, patients will benefit from support along the journey of chronic care management. Given the potential for telemedical interventions to be clinically effective in improving diabetes control overall and significantly improving HbA_1c_ concentrations [44], it is vital that these strategies be optimized to address potential challenges in advance, ensuring quality of care and value in health.

Understanding the experiences of patients with diabetes and clinicians who care for populations with diabetes is important, especially given the significant risks that patients with diabetes face due to infectious diseases such as COVID-19 [8]. The ability to avoid facility waiting rooms, offices, and exposure to large numbers of people makes telehealth an important tool for people with diabetes, and this factor coupled with the dramatic increase in the use of telehealth services in recent years [11] makes it crucial to investigate lived experiences with telehealth. Some of this study’s findings may be representative of an abrupt change to telehealth; for example, the facilitator of increased safety and comfort of telehealth during the pandemic and technological challenges. However, other themes are likely to be more persistent, such as the downside of convenience and mismatched expectations of telehealth visit efficiency and wait times. Additional studies should focus on whether barriers to telehealth seen in the immediate period after the declaration of the COVID-19 pandemic are persistent for people with diabetes and their health care providers or whether these fade for patients and clinicians.

### Strengths and Limitations

This study used a rigorous qualitative approach to understand the telehealth experiences of patients living with diabetes during COVID-19 as well as the experiences of clinicians who treat patients with diabetes in Louisiana. This study had some limitations. First, the participants were mostly older adults, which may not be fully representative of all populations with diabetes who use telehealth, but were representative of key populations of interest in the region with the largest burden of chronic disease. Clinicians from a range of ages and years of practice were included. Different characteristics and experiences did provide a range of perspectives on telehealth barriers and facilitators. Second, some challenges were encountered in recruitment, including pandemic shutdowns and a hurricane in the region, which may have affected which participants were ultimately able to participate in the study. Finally, participant reports of experiences may have been influenced by recall bias or social desirability bias. Interviews took place at different lengths of time from the participants’ last experiences with telehealth. Certain limitations were outside our control (pandemic and hurricane); however, efforts to ensure reliability and trustworthiness were used throughout the study. As with all studies, there is a risk that participants may selectively present positive and socially desirable responses to experiences.

### Conclusions

Telehealth plays an important role in the management of diabetes and may be especially important in areas with high prevalence, such as Louisiana. Issues of internet connectivity and proficiency using technology must be addressed to ensure equitable access across patient populations. In addition, support to help guide and inform patients on what to expect and how to prepare for telehealth visits is recommended to improve the experience of both health systems and patients. Preparing patients with diabetes for what to expect during telehealth visits, including having important health information available during the visit and managing expectations over wait times, is an important strategy. Similarly, allowing clinicians to schedule telehealth and in-person visits during discrete blocks of time could encourage efficiency. Further research should address how hybrid models of telehealth and in-person care may differentially impact health outcomes for patients with diabetes, particularly for people with multiple chronic conditions and in settings where access to technology and connectivity is not optimal.

## Abbreviations

HbA1c: hemoglobin A1c

## Data Availability

All data generated or analyzed during this study are included in this published paper.
